# Virtual Reality Training for Balance in Patients with Chronic Low Back Pain: A Systematic Review and Meta-Analysis

**DOI:** 10.3390/jcm14207247

**Published:** 2025-10-14

**Authors:** Abrar I. AlSadiq, Fuad A. Abdulla, Ali M. Alshami

**Affiliations:** 1 Department of Physical Therapy, Dammam Medical Complex, Dammam 32253, Saudi Arabia; 2 Department of Physical Therapy, Faculty of Allied Medical Sciences, Philadelphia University, Amman 19392, Jordan; fabdulla@philadelphia.edu.jo; 3 Department of Physical Therapy, College of Applied Medical Sciences, Imam Abdulrahman Bin Faisal University, Dammam 31441, Saudi Arabia; alshami@iau.edu.sa

**Keywords:** dynamic, rehabilitation, randomized clinical trial, static, video games

## Abstract

**Background:** Chronic low back pain is often associated with impaired balance and reduced functional mobility. Recent studies suggest that virtual reality-based interventions may be effective in improving balance outcomes in individuals with chronic low back pain. **Objective:** In this systematic review and meta-analysis, we aimed to investigate the impact of virtual reality training on static and dynamic balance outcomes in patients with chronic low back pain. **Methods:** Two independent reviewers searched English-language studies from inception to 1 July 2024, using the following databases: PubMed, Web of Science, Scopus, Dimensions, Semantic Scholar, and ProQuest. Randomized clinical trials with a PEDro score of ≥6 were included. Fixed- and random-effects meta-analyses were conducted on eligible trials. **Results:** Of 3172 records screened, 13 trials were eligible. Meta-analyses of six trials (*n* = 183) across diverse adults using 2–8 week interventions showed that virtual reality training improved dynamic balance: timed up and go (mean difference: −2.29 s; 95% confidence interval: −2.91 to −1.66; I^2^ = 0%; *p* < 0.00001) and forward reach (mean difference: 7.80 cm; 95% confidence interval: 2.08 to 13.52; I^2^ = 0%; *p* = 0.008). However, no significant effects were found for static balance, single-leg stance, center of pressure medio-lateral displacement, or center of pressure velocity, compared with controls. **Conclusions:** Virtual reality-based training seems to be more effective than control interventions in improving dynamic and functional balance, but not static balance, in patients with chronic low back pain.

## 1. Introduction

Low Back Pain (LBP) is one of the most prevalent musculoskeletal conditions globally, with up to 80% of individuals experiencing at least one episode during their lifetime [1]. Approximately 10–40% of those affected by LBP eventually develop chronic LBP(CLBP) [2]. Numerous studies have associated CLBP with impaired lumbosacral proprioception and diminished balance performance [3,4]. Altered sensory discrimination resulting from increased stress on the proprioceptive systems has been linked to changes in postural sway control in individuals with CLBP compared to healthy controls [5].

Clinical practice guidelines for managing CLBP emphasize non-invasive, patient-centered approaches. For instance, the European guidelines recommend a multimodal approach, including structured exercise therapy and biopsychosocial interventions such as cognitive behavioral therapy [6]. Similarly, the North American Spine Society guidelines from 2020 recommend conservative treatments such as patient education and non-opioid medications [7,8]. New technologies, such as virtual reality (VR), have demonstrated improvements in pain intensity, pain-related fear [9,10], and disability [11] in patients with CLBP. Preliminary systematic reviews have also suggested that VR could have positive effects on balance and gait in healthy older adults [12], as well as in patients with Parkinson’s disease [13] and multiple sclerosis [14]. VR has demonstrated rehabilitative benefits in these neurological populations, primarily through mechanisms that enhance sensorimotor integration, attentional engagement, and motor learning [15]. For example, a study reported that VR-based gait training improved mobility and reduced fall risk in Parkinson’s disease patients [16], while another showed that VR interventions enhanced balance and functional outcomes in individuals with multiple sclerosis [17]. These mechanisms plausibly translate to CLBP by targeting impaired postural control, fear-avoidance behaviors, and reduced movement variability. Individual clinical studies have explored the effects of VR on balance in people with LBP and have demonstrated varying results [18,19,20]. These findings indicate VR’s potential as a balance rehabilitative tool across neurological and musculoskeletal conditions. However, for patients with chronic low back pain (CLBP), the available evidence remains limited and inconsistent, particularly regarding static and dynamic balance outcomes. Therefore, this review aims to systematically synthesize randomized controlled trial evidence to clarify the role of VR-based training in balance rehabilitation for CLBP.

## 2. Materials and Methods

This review was conducted according to the Preferred Reporting Items for Systematic Reviews and Meta-Analyses (PRISMA) guidelines [21] and was registered in the International Prospective Register of Systematic Reviews (PROSPERO) on 9 February 2023, No. CRD42023395264.

### 2.1. Search Strategy

Two reviewers (AIS and FAA), working independently, searched PubMed, Ovid (MEDLINE), ProQuest, Dimensions, Scopus, Semantic Scholar, and Web of Science (WoS). All databases were searched from inception until 1 July 2024 to identify relevant articles. The following keywords were generated by a medical librarian: “LBP,” “Balance,” “postural stability,” “COP,” and “Virtual Reality.” In PubMed, a search using Medical Subject Headings (MeSH) was also conducted using the term “Virtual Reality AND LBP*.” Full details of the search strategy are provided in Supplementary File S1. Furthermore, the reference lists of included studies and relevant reviews were screened.

### 2.2. Study Selection

The Population, Intervention, Comparison, Outcomes, and Study design (PICOS) framework was used to guide study inclusion. Participants were adults (≥18 years) with CLBP lasting at least 3 months. The intervention consisted of VR-based training or exercise. Included studies were required to apply at least one outcome measure related to balance and/or stability (e.g., single leg stance [SLS] test, center of pressure [CoP] deviations, or comparable measures). Only randomized controlled trials (RCTs) with pre- and post-intervention measurements were included. Articles were excluded if they were gray literature, conference abstracts, reviews, not available in full text, or not published in English. Duplicate records were removed using Covidence systematic review software (Veritas Health Innovation, Melbourne, Australia). The access date was 1 July 2024 at: https://app.covidence.org. Then, the reviewers (AIS and FAA), working independently, screened the titles and abstracts for eligibility. A second screening of the full texts was performed by the same reviewers. In cases of disagreement, conflicts were resolved through online discussion to reach consensus.

### 2.3. Quality Assessment and Risk of Bias

The two reviewers (AIS and FAA) independently evaluated the quality and risk of bias of the trials using the physiotherapy evidence database (PEDro) scale [22]. In cases of discrepancy between reviewers, an online meeting was held to reach a final decision. Overall evaluation was based on PEDro scores to assess the internal and external validity of each study for inclusion [22]. Assessment criteria included eligibility, randomization, and allocation concealment, baseline group similarities, blinding (of patients, assessors, and therapists), dropouts, between-group comparisons, and intention-to-treat analysis [22]. PEDro scores of 0–4 were classified as ‘poor’, 4–5 as ‘fair’, 6–8 as ‘good’, and 9–10 as ‘excellent’. A total PEDro score of ≥7 indicated a lower risk of bias compared to scores ≤ 3 [23]. Only trials with PEDro scores ≥ 6 were considered for inclusion in the review [22].

### 2.4. Evidence Certainity Assessment (GRADE)

To evaluate the overall certainty of evidence, we applied the Grading of Recommendations Assessment, Development and Evaluation (GRADE) approach across the five balance-related outcomes included in this review. The GRADE framework considers study design, risk of bias, inconsistency, indirectness, imprecision, and publication bias to classify the certainty of evidence into four levels: high, moderate, low, or very low [24].

### 2.5. Data Extraction and Synthesis

The two reviewers (AIS and FAA) independently retrieved all required data using an extraction table. Discrepancies were resolved by consensus through discussion. Selected studies were synthesized based on the following variables: authors (year of publication), study design, country, population group (*N*) and subgroups (*n*), percentage of male participants, mean age and standard deviation, type of virtual environment (level of immersion), device (type of feedback), treatment, treatment period (dose and frequency), balance outcome measure(s), main finding(s), and PEDro score.

## 3. Statistical Analysis

Statistical heterogeneity was evaluated by calculating I^2^ [25]. I^2^ values of <50% were considered for a fixed-effects analysis model, whereas values ≥ 50% were considered indicative of a random-effects model [26]. Five separate meta-analyses were conducted to investigate the effect of interventions on balance outcome measures. Due to excessive divergences among the extracted outcome measures, a meta-analysis could not be conducted to differentiate the effects between varying levels of immersion (low vs. moderate vs. high) within the experimental group. Publication bias was difficult to assess in balance outcome measures using funnel plots due to the small number of studies included in each analysis (maximum of three). As an alternative assessment of publication bias, a trial registry check was performed using ClinicalTrials.gov to identify any unpublished studies meeting the inclusion criteria for the meta-analysis. A meta-analysis for every subgroup was performed on only six RCTs, and the pooled mean difference (MD) was calculated using the inverse variance (IV), fixed-effect model, 95% confidence interval (CI), and *p*-value. Review Manager (RevMan) software version 5.4.1, 2020 (Cochrane Collaboration, Copenhagen, Denmark) was used for the meta-analysis and to generate forest plots.

## 4. Results

### 4.1. Article Selection

**Figure 1:**
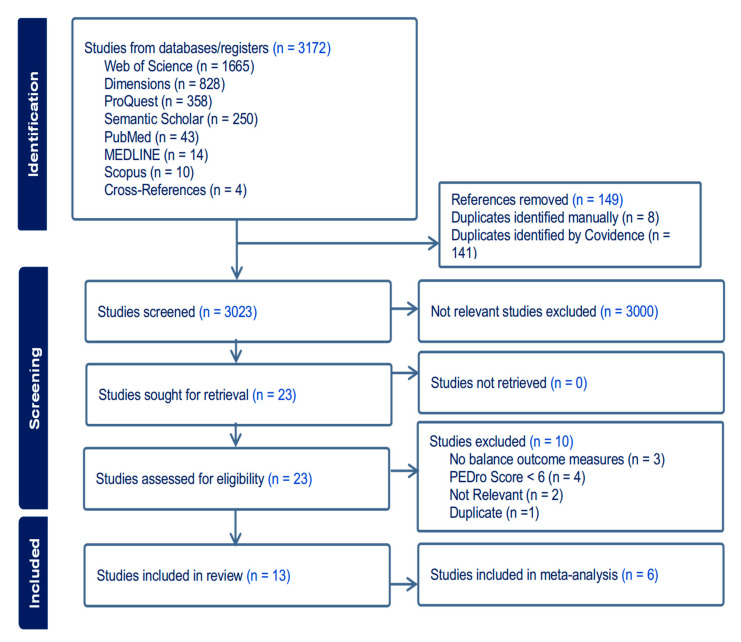
Flowchart of study selection according to PRISMA. RCT: randomized clinical trial.

The search strategy identified 3172 studies (Figure 1). Of these, 149 records were removed as duplicates, and 3000 were excluded based on title and abstract irrelevance. Twenty-three studies were eligible for full-text review. Of these, 13 RCTs were included in this systematic review [18,20,27,28,29,30,31,32,33,34,35,36,37]. A full table of excluded articles at this stage can be found in Supplementary File S2. Only six of the included RCTs were eligible and utilized in the meta-analysis [18,20,27,31,32,35]. The trial registry search did not yield any eligible unpublished studies that matched the inclusion criteria, and all 10 resultant studies were excluded (Supplementary File S3).

### 4.2. Study Characteristics

A total of 365 participants were involved in the 13 included RCTs (174, 171, and 20 in experimental, control, and other comparative groups, respectively). Slightly more than half of the participants (185, 50.7%) were women. The ages of the participants across the included studies ranged from 18 to 78 years. The frequency of intervention ranged from one to five sessions per week, lasting from 1 to 12 weeks in total. All RCTs were published in English between 2013 and 2024. Each experimental treatment program included a VR-based regimen with varying levels of immersion: 69% and 31% of RCTs employed moderate [27,28,29,30,31,32,34,36,37] and high immersion levels [18,33,35], respectively. VR interventions included horse riding, balance-board training, core stability training, postural stability training, limits of stability (LoS) training, weight-shift training, maze control training, and a wide variety of games. These VR interventions were compared to control interventions involving conventional physical therapy [27,28,31], no intervention [32,33,35,37], strength and/or core stability training [18,27,29,30], balance training [20], magnetic therapy (MT) [34], or other variations in VR training [36]. Seven RCTs applied treatment sessions over the short term (≤4 weeks) [20,30,31,32,34,36,37], and six RCTs applied them for a more extended period (>4 weeks) [18,27,28,29,33,35] (Table 1). All CoP directional-displacement and velocity parameters were evaluated using plantar pressure platforms in two RCTs during static standing balance measurements—one with eyes closed [32] and one with eyes open [35].

### 4.3. Characteristics of Meta-Analysis Studies

Six randomized controlled trials (*n* = 183) tested virtual reality (VR)–enhanced exercise for chronic or nonspecific low back pain. Participants ranged from young collegiate male athletes [18] to older women [35]; about 60% overall were female [20,27,31]. VR delivery included immersive headsets with core stability training [18], Nintendo Wii exergames [27], passive VR walking [31], home trunk-feedback exergaming [32], and laboratory VR balance platforms [20]. Controls were core stability alone [18], conventional physiotherapy [31], lumbar stabilization [27], or no intervention [32]. Programs lasted 2–8 weeks, usually 3 sessions/week (20–45 min) [27,35]. Outcomes spanned pain, disability, kinesiophobia, postural sway/balance, and quality of life [20,27,31,32]. Most studies reported greater improvement in balance, pain, and function with VR adjuncts [18,31,35], while smaller pilots showed variable effects [20,32].

### 4.4. Quality and Risk of Bias Assessment

The mean PEDro score of all RCTs was 7.4 out of 10 (range: 6–9), indicating good quality (Table 2). While all 13 RCTs reported random allocation, baseline comparability, between-group results, and measures of variability, only seven trials addressed allocation concealment. Participant blinding was reported in one RCT, therapist blinding in five, and assessor blinding in seven RCTs. One RCT failed to retain data from >85% of initially included participants at allocation, and one RCT did not meet the criterion of either receiving treatment as allocated or performing an intention-to-treat analysis.

**Table 1:** Characteristics of included randomized clinical trials.

Author, Year	Country	Population Group (*N*): Subgroups (*n*), % Male	Age (Mean ± SD)	Environment (Level of Immersion)	Device (Type of Feedback)	Intervention	Dose	Balance Outcome Measure(s)	Main Findings
Abdelraouf et al., 2020 [18]	Egypt	NSLBP in collegiate male athletes (50): EG (25) CG (25), 100%	EG: 20.9 ± 5.2 CG: 22.1 ± 2.6	Lightweight headset with 2 games: (No Limits 2 Roller Coaster) and (Euro Truck 2 simulations). (high)	Oculus Rift DK2 (audiovisual)	EG: core stability exercises and virtual reality CG: core stability exercises	6 weeks (2 sets × 20 repetitions with 15 s hold for each exercise, 5 days/week)	Dynamic Balance (SEBT)	EG was significantly better than CG in anterior (*p* = 0.031), posterolateral (*p* = 0.034), and posteromedial (*p* = 0.037) directions.
Chen et al., 2016 [30]	Korea	NSLBP (19): EG (10) CG (9), NIA	Overall: between 19 and 30 years EG: NIA CG: NIA	VR-based horse-riding simulator (moderate)	Indoor riding machine (Hongjin Leports) and 2D screen (visual, motion haptic)	EG: lumbar strengthening exercise (15 min) + horse riding simulator exercise (15 min). CG: lumbar strengthening exercise (30 min)	4 weeks (12 sessions of 30 min)	Forward and backward LoS	Significant improvement in both groups (*p* < 0.05). No significant difference between the groups (*p* > 0.05)
Cikajlo et al., 2016 [20]	Slovenia	CLBP expanding into the lower limb (11): EG (6) CG (5), 27.3%	EG: 56.8 ± 12.4 CG: 56.8 ± 12.4	Graphical computing environment with ball rolling on virtual path + weight (COG) shift (moderate)	Gamma trainer (PHU Technomex Sp., Gliwice, Poland (visual)	EG: balance training with Gamma device. CG: balance training with a wobble board	2 weeks (5 consecutive days/week)	Postural perturbation response (overshoot and latency) Functional reaching Single Leg standing	No significant differences between both groups (*p* > 0.05).
Li et al., 2021 [34]	China	CNLBP (34): EG (11) CG (11) MCE (12), 26.47%	EG: 21.9 ± 2.4 CG: 25.4 ± 3.7 MCE: 23.8 ± 4.1	VR-based Kinect games: Fruit Ninja game (moderate)	Kinect Xbox 360 systems (audiovisual)	EG: thermal magnetic therapy + VR training CG: thermal magnetic therapy MCE: Thermal magnetic therapy + MCE training	EG: 5 weeks (MT + 6 game sessions/day × 5 days/week) CG: two weeks (20 min × 5 days/week). MCE: 5 weeks (10 repetitions × 3 sessions of ultrasound-guided ADIM + 4-point kneeling 5 days/week).	Activation Time: APAs CPAs	EG: MF muscle activated later (*p* = 0.001) Significant decrease in TrA (*p* = 0.002) and TA (*p* = 0.007) muscle activity during 1st CPA. The IEMGs of TrA (*p* = 0.002) and TA (*p* = 0.007) during 1st CPA CG: No significant changes (*p* > 0.05).
Meinke et al., 2022 [32]	Switzerland	NSLBP (24): EG (13) CG (14), 37%	EG: 40.9 ± 15.2 CG:40.1 ± 12.4	VR-based ValedoMotion Home system (moderate)	Wireless motion sensors Valedo Pro (Hocoma AG), 2D screen (Audiovisual)	EG: movements of the upper body or the pelvis CG: no intervention	3 weeks (9 sessions of 20 min)	CoP during quiet standing: Medial-lateral displacement Global displacement Anterior–posterior velocity Medial-lateral velocity Global velocity	No changes in any outcomes in any group (*p* ≥ 0.25)
Monteiro-Junior et al., 2015 [29]	Brazil	CLBP (30): EG (16) CG (14), 0%	Overall: 68 ± 4 EG: NIA, CG: NIA	VR-based Nintendo Wii Fit program (moderate)	Nintendo Wii Motion Tracking System/2D screen (Wii balance board (audiovisual)	EG: lower limb strength exercises + core training + Wii-based exercise CG: lower limb strength exercises + core training.	8 weeks (3×/week) EG: strengthening and core 10–15 s ×3; +30 min of virtual physical training CG: strengthening and core 10–15 s ×3	Static balance (Wii balance board measuring (EA) of displacement of (CoP) (cm^2^)	No significant within or between groups differences in balance (*p* > 0.01)
Mueller et al., 2022 [36]	Germany	CLBP (13): cross-over trial: EG-CG (7) CG-EG (6), 38.5%	EG-CG: 2 males: 26.5 ± 4 years, five females: 50 ± 13 years CG-EG: 3 males: 41 ± 25 years, 3 females: 34 ± 11 years	VR-home based (Valedo Home; Hocoma, Switzerland) (moderate) avatar movement in 3 levels (moderate)	Valedo Home System + application-based software with tablet/smartphone (audiovisual)	EG-CG: trunk movements training ⟶ rest CG-EG: rest ⟶ trunk movements training	One session: 12 min intervention and the 12-min-rest-time	Proprioception (angle reproduction)	No significant change in angle reproduction between both groups (*p* > 0.05)
Park et al., 2013 [27]	South Korea	Work related CLBP (24): EG (NWE) (8) Second intervention group of LSE (8); CG (8), 100%	NWE: 44.1 ± 5.5 LSE: 43.4 ± 5.4 CG: 45.5 ± 5.3	VR-based Nintendo Wii Sports games (moderate)	Nintendo Wii Motion Tracking System/2D screen (audiovisual)	NWE: Conventional + Nintendo Wii exercise program, including wakeboard, Frisbee dog, jet ski, and canoe games LSE: conventional + stabilization exercise CG: conventional only.	8 weeks (each session: 50 min conventional (all groups) and 30 min exercise program (LSE and NWE) × 3 times/week)	Functional balance (One-legged stand test)	Only CG and LSE groups improved significantly (*p* < 0.05).
Suh et al., 2018 [37]	Korea	CLBP (20): EG (10) CG (10), 0%	EG: 72.3 ± 5.3 CG: 66.7 ± 3.1	VR-based Nintendo Wii sports games (moderate)	Nintendo Wii Motion Tracking System/2D screen (audiovisual)	EG: Wii Sports Tennis, Bowling, and Golf CG: no exercises	4 weeks (30 min × 3 times/week)	Berg balance scale	No significant difference between both groups (*p* > 0.05)
Tomruk et al., 2020 [28]	Turkey	CLBP (42): EG (21) CG (21), NIA	Median (IQR) EG: 46 (40.05–50.50) CG: 45 (44–48)	Computer-based stability training (moderate)	Biodex Balance System (visual)	EG: Postural stability, limits of stability, weight shift, and maze control training. CG: Traditional postural training exercises.	12 weeks (2 times a week; 30 min/session).	Postural control (LoS and PS tests by Biodex Balance System)	Both groups improved significantly (*p* < 0.05), but EG showed a better effect than CG (*p* = 0.023).
Yalfani et al., 2022 [33]	Iran	CLBP (27): EG (13) CG (14), 0%	EG: 68 ± 2.9 CG: 67.1 ± 2.9	Adrenaline station game center (high)	HTC Vive virtual reality system (audiovisual)	EG: Fisher, Boxing, Tennis, Football, Bowling, Beat Saber, Audio shield, and Skiing) VR training. CG: no intervention	EG: 8 weeks (3 sessions/week, with 30 min for each session) CG: Daily routine.	Fall Risk Index	EG improved better than CG (*p* = 0.001)
Yalfani et al., 2024 [35]	Iran	CLBP (24): EG (12) CG (12), 0%	EG: 68.3 ± 2.9 CG: 67.1 ± 2.9	Adrenaline Station Game Center (high)	HTC-Vive virtual reality headset system (audiovisual)	EG: Training package 1: fish-catching, boxing, tennis, and football. Training package 2: boxing, skiing, bowling, Beat Saber, and Audio-shield. CG: No rehabilitation	EG: 8 weeks (3× 30 min training sessions/week)	Plantar pressure CoP fluctuations (anterior–posterior, medial-lateral, and sway velocity) -TUG	EG improved in all outcomes compared to CG (*p* ≤ 0.02)
Yelvar et al., 2017 [31]	Turkey	NSLBP (44, 36.4%): EG (22, 54.5%) CG (22, 18.2%).	EG: 46.3 ± 3.4 CG: 52.8 ± 11.5	Video clip in natural walking down Ireland forest at a speed of 1.0 km/h (moderate)	iPod (Apple Inc., Apple Park, CA, USA) with video glasses (Wrap920, Vuzix Corporation) (visual)	EG: virtual walking + traditional physiotherapy CG: traditional physiotherapy	5×/2 weeks	Single-Leg Balance Test TUG	No significant difference in single-leg balance between groups (*p* = 0.099). EG improved better than CG in TUG (*p* < 0.001).

SD: standard deviation, CLBP: chronic low back pain, EG: experimental group, CG: control group, COG: center of gravity, NSLBP: nonspecific low back pain, s: second, SEBT: Star excursion balance test, NWE Nintendo Wii exercise, LSE: Lumbar stabilization exercise, VR: Virtual Reality, NIA: no information available, EA: Elliptical area, IQR: interquartile range, LoS: limits of stability, PS: postural stability, TUG: time-up-go test, MCE: motor control exercise, ADIM: abdominal drawing-in maneuver, APAs: anticipatory postural adjustments, CPAs: compensatory postural adjustments, TrA: transverse abdominis, MF: multifidus, TA: tibialis anterior, IEMGs: integrals of the electromyography activities.

**Table 2:** Physiotherapy Evidence Database (PEDro) scores of included randomized clinical trials.

Study	1. Random Allocation	2. Concealed Allocation	3. Baseline Comparability	4. Blinding Subjects	5. Blinding Therapists	6. Blinding Assessor	7. Outcome Data > 85%	8. Condition as Allocated or Intention to Treat	9. Between-Group Results	10. Variability Measures	Total
Abdelraouf, 2020 [18]	1	1	**1**	0	1	0	1	1	1	1	8/10
Chen, 2016 [30]	1	0	1	0	0	0	1	1	1	1	6/10
Cikajlo, 2016 [20]	1	0	1	0	0	0	1	1	1	1	6/10
Li, 2021 [34]	1	0	1	0	1	1	1	1	1	1	8/10
Meinke, 2022 [32]	1	1	1	0	1	1	1	1	1	1	9/10
Monteiro-Junior, 2015 [29]	1	1	1	0	0	1	1	1	1	1	8/10
Mueller, 2022 [36]	1	1	1	1	1	1	1	0	1	1	9/10
Park, 2013 [27]	1	0	1	0	0	0	1	1	1	1	6/10
Suh, 2018 [37]	1	0	1	0	0	0	1	1	1	1	6/10
Tomruk, 2020 [28]	1	1	1	0	0	1	1	1	1	1	8/10
Yalfani, 2022 [33]	1	1	1	0	0	0	1	1	1	1	7/10
Yalfani, 2024 [35]	1	1	1	0	1	1	0	1	1	1	8/10
Yilmaz Yelvar, 2017 [31]	1	0	1	0	0	1	1	1	1	1	7/10

### 4.5. Proof of Efficacy

All 13 included RCTs had at least one outcome measure that quantitatively evaluated balance. Balance outcomes varied greatly between studies. These outcomes included postural perturbation response, postural control (e.g., LoS and postural stability with CoP fluctuations), functional reaching (e.g., star excursion balance test [SEBT]), static balance parameters measured by the Wii Balance Board or the SLS test, timed-up-and-go (TUG), fall risk index, activation time of anticipatory and compensatory postural adjustments, proprioception (e.g., angle reproduction), and the Berg Balance Scale. Meta-analyses were applicable to only five outcome measures: SLS test, CoP mean medio-lateral displacement, CoP velocity changes, TUG, and dynamic balance in functional reaching.

#### 4.5.1. Single Leg Stance

**Figure 2:**
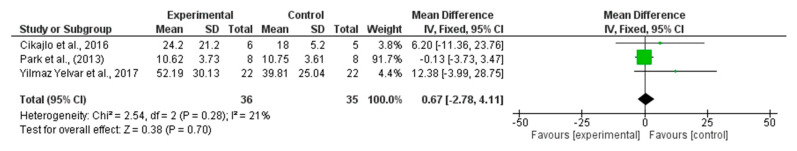
Forest plot of single leg stance time comparing VR-based training with control interventions in individuals with chronic low back pain (0–50 scale) [20,27,31].

Based on three RCTs [20,27,31], meta-analysis showed that VR-based training did not improve SLS better than control interventions (mean difference [MD]: 0.67 s; 95% CI: −2.78 to 4.11; I^2^ = 21%) (Figure 2).

#### 4.5.2. CoP Mean Medio-Lateral Displacement

**Figure 3:**
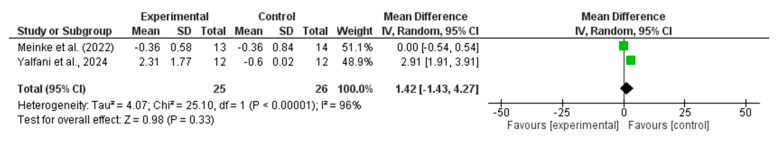
Forest plot of Center of Pressure mean medio-lateral displacement comparing VR-based training with control interventions in individuals with chronic low back pain (0–50 scale) [32,35].

Based on two RCTs [32,35], VR-based training did not demonstrate a superior effect on CoP mean medio-lateral displacement (MD: 1.42 mm; 95% CI: −1.43 to 4.27; I^2^ = 96%) (Figure 3).

#### 4.5.3. CoP Velocity

**Figure 4:**
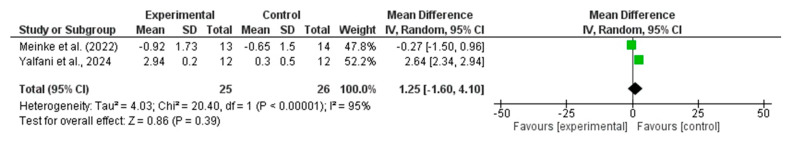
Forest plot of Center of Pressure velocity comparing VR-based training with control interventions in individuals with chronic low back pain (0–50 scale) [32,35].

Based on two RCTs [32,35], VR-based training did not demonstrate a greater effect on CoP velocity compared to the control interventions (MD: 1.25 mm/s; 95% CI: −1.60 to 4.10; I^2^ = 95%) (Figure 4).

#### 4.5.4. Timed Up and Go (TUG)

**Figure 5:**
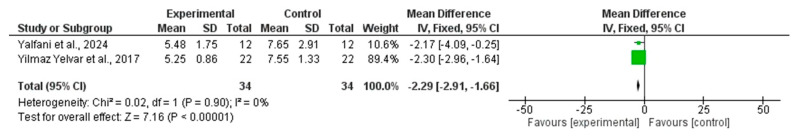
Forest plot of Timed Up and Go (TUG) time comparing VR-based training with control interventions in individuals with chronic low back pain (0–50 scale) [31,35].

Based on two RCTs [31,35], VR-based training reduced TUG scores compared to the control interventions (MD: −2.29 s; 95% CI: −2.91 to −1.66; I^2^ = 0%) (Figure 5).

#### 4.5.5. Dynamic Balance in Reaching

**Figure 6:**
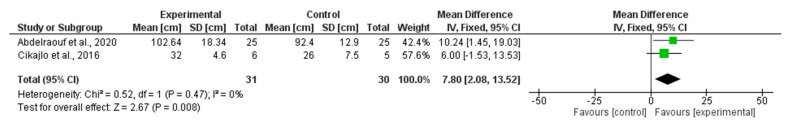
Forest plot of dynamic balance during functional reaching comparing VR-based training with control interventions in individuals with chronic low back pain (0–50 scale) [18,20].

Based on two RCTs [18,20], VR-based training demonstrated improved dynamic balance during reaching tasks compared to the control interventions (MD: 7.80 cm; 95% CI: 2.08 to 13.52; I^2^ = 0%) (Figure 6).

### 4.6. Evidence Grading for Key Outcomes

The GRADE evaluation indicates moderate confidence in the evidence supporting improvements in dynamic balance outcomes, whereas static balance outcomes are supported by very low to low confidence, largely due to high heterogeneity, small sample sizes, and limited trials. Detailed certainty ratings for each outcome are presented in Table 3.

## 5. Discussion

This systematic review and meta-analysis assessed the efficacy of VR-based training on several balance outcomes in individuals with CLBP. The review incorporated 13 RCTs, each evaluating at least one quantitative measure of balance. Outcomes varied across studies, and the meta-analysis of six eligible RCTs included the following outcomes: static balance (e.g., SLS), dynamic balance (e.g., SEBT and functional reaching test), functional mobility (e.g., TUG), and postural control metrics (e.g., CoP displacement and velocity). Significant improvements were observed in TUG scores and functional reaching tests in favor of VR interventions, while no significant effects were found for SLS, CoP medio-lateral displacement, or CoP velocity. The findings suggest that VR-based training can positively influence dynamic and functional aspects of balance in individuals with CLBP.

The lack of significant findings in static balance measures such as SLS and CoP metrics may reflect limitations in the sensitivity of these tests to detect meaningful changes, or it may suggest that the benefits of VR are more pronounced during dynamic and task-specific activities [38]. The high heterogeneity observed in some meta-analyses (e.g., I^2^ = 96% for CoP displacement) could relate to variability in study designs, VR modalities, and participant populations, which may have diluted the overall effect sizes. Despite the lack of achieved effects in our analysis, SLS has previously been demonstrated to be a valid measure of static balance deficiencies in LBP [39]. However, deficiencies in static balance have been more linked in the literature to CLBP through various associations with pain characteristics, lumbar function, and psychological contributions, giving these factors a particularly significant value to be considered in the assessment of static balance [40].

The significant reduction in TUG time indicates enhanced mobility and functional balance, while improvements in reaching tasks highlight better postural control during goal-directed movements. These effects may be attributed to the interactive and immersive nature of VR, which encourages engagement, motor planning, and multisensory integration [41]. In CLBP predominantly, TUG is reportedly a beneficial tool that can provide sufficient information regarding the dynamics of kinematic adaptations in temporal parameters [42].

Relatedly, dynamic balance outcomes (SEBT and functional reaching) improved significantly as well, supporting the effectiveness of interventions in enhancing dynamic postural control during goal-directed movements [41]. The VR improvements may have stem from the immersive and task-oriented nature of its environments, which likely stimulate multisensory integration, motor planning, and real-time feedback processing reactive mechanisms critical for dynamic balance. This was expected, because dynamic balance insufficiencies were clearly reported in patients with CLBP [43,44].

Several studies corroborate the positive impact of VR on dynamic balance and functional mobility in different populations. For instance, an RCT concluded that VR therapy was more effective than conventional balance exercises in enhancing dynamic balance, cognitive-motor abilities, and reducing falls in people with multiple sclerosis [17]. Moreover, VR therapy yielded better outcomes compared to traditional balance training and physical exercise in this population [17]. Likewise, a recent meta-analysis revealed that VR training significantly improved functional mobility and balance in older adults with balance impairment compared to traditional physical training. The study highlighted that VR with longer intervention sessions and higher frequency induced significant benefits, in line with similar findings in the same population [45]. Conversely, some previous evidence has presented contrasting findings. For example, a systematic review and meta-analysis of RCTs indicated that while non–head-mounted VR had beneficial effects on standing balance in older adults, it did not demonstrate significant improvements in gait, mobility, or fear of falling [46]. A 2022 scoping review revealed that the effect of VR on function was inconsistent in patients with CLBP [47]. While some studies reported enhancements in physical function, such as improved mobility, posture, or task-specific performance, others showed no significant change. The variability in outcomes was attributed to differences in VR content, intervention duration, outcome measures, and sample characteristics [47].

Further, regarding the point of VR training significantly improving timed mobility, as reflected in reduced TUG completion times [31,35], this aligns with the theoretical framework that VR’s enriched, goal-directed feedback enhances anticipatory postural adjustments (APAs) and cognitive–motor coupling, particularly for tasks requiring ongoing weight-shifting and stepping strategies [34]. Functional reaching followed the same pattern as the analysis of both included RCTs demonstrated an advantage for VR training over controls [18,20], suggesting that reaching tasks, while dynamic in nature, could rely more heavily on task familiarity, biomechanical efficiency, and motor repetitions, which may be better addressed through VR environments than through conventional approaches [48]. By contrast here, static posture outcomes (e.g., quiet stance) remained generally less responsive to VR training, likely due to ceiling effects and the limited challenges of simple stance conditions, in addition to the short intervention durations reported across our included trials and supported by previous ones [49]. Notably, our observed improvement in TUG time (2.29 s) exceeds published minimal clinically important difference (MCID) thresholds reported for lumbar spine populations (approximately 2.1–3.4 s), supporting the clinical relevance of these mobility gains [50,51].

One last point in regards of a main contributor to the achieved results consideration; heterogeneity. Heterogeneity levels varied considerably across outcomes as our GRADE analysis has clearly shown. For instance, heterogeneity in SLS was low (I^2^ = 21%), suggesting that differences between trials were minimal, although variations in VR modality (Kinect vs. immersive systems) and participant characteristics may still have influenced results. In contrast, both CoP medio-lateral displacement (I^2^ = 96%) and CoP velocity (I^2^ = 95%) displayed extreme heterogeneity, likely due to marked differences in immersion levels and intervention dosages, with some studies applying short, low-frequency programs while others implemented more extended protocols. Age-related variability across populations may also have contributed, as younger and older participants differ in neuroplasticity and postural control responsiveness. By comparison, outcomes such as Timed Up and Go (I^2^ = 0%) and forward reaching (I^2^ = 0%) demonstrated no heterogeneity, indicating that mobility and functional balance measures may be more consistently influenced by VR interventions, regardless of modality or dosage. While the results for mobility (TUG) and functional reach are robust, outcomes related to CoP measures were influenced by individual study and intervention variability. For instance, both included trials assessed mediolateral CoP excursion during static posturography. One study recorded CoP displacement on a force platform with participants standing quietly with eyes closed [32], whereas the other study derived mediolateral fluctuations using a plantar-pressure system under similar standard quiet-standing conditions [35]. Consequently, the observed high heterogeneity (I^2^ = 96%) cannot be attributed to a static–dynamic contrast. Instead, the variation more plausibly reflects differences in sensory conditions (eyes closed vs. likely eyes open), measurement devices (force platform vs. plantar-pressure mat), and sample characteristics (adults with low back pain vs. older women). These methodological contrasts may explain the opposing study effects despite both trials evaluating static balance. The findings for static balance (SLS) appear relatively stable, though limited by small sample sizes. These observations reinforce the need for larger, more standardized trials to reduce heterogeneity in future research.

Generally, inconsistency in the findings may largely be attributed to variations among the included RCTs. For example, the diversity of VR systems and training protocols limited the comparability of results across studies. In addition, the small number of studies eligible for each meta-analysis reduced the statistical power and generalizability of the findings. Moreover, some studies had small sample sizes and short intervention durations, which may have influenced the magnitude and sustainability of the effects. Furthermore, populations across the studies were highly diverse, particularly in age, which likely contributed to variability in the underlying causes of CLBP, because these tend to differ between younger and older individuals.

Collectively, the findings highlight that while VR-based training shows robust effects on certain functional outcomes, static and CoP-related measures are more sensitive to variability in intervention characteristics and participant profiles, warranting greater standardization in future trials. Future studies should therefore tailor VR interventions to address both static and dynamic domains: incorporating static-balance-specific challenges (narrowed base, eyes-closed, dual-task paradigms) and refining dynamic tasks to more closely mimic real functional demands.

## 6. Strengths and Limitations

A key strength of this review is the inclusion of only RCTs with high-quality PEDro scores, enhancing the methodological rigor of the findings. Additionally, the review incorporated a broad range of balance outcomes, capturing both static and dynamic aspects of balance performance. The main limitation of this meta-analysis was the substantial heterogeneity in balance outcome measures, which reduced comparability across trials. Restricting inclusion to English-language publications and excluding all grey literature may have introduced selection bias, while the small number of studies (<10 in each analysis) limited the ability to assess publication bias properly. In addition, variability in hardware, content, and dosage further constrained the certainty of the findings. Effect sizes should therefore be interpreted cautiously, which underscores the need for adequately powered RCTs.

## 7. Clinical Implication

The disparity in the effects of VR between static and dynamic balance outcomes highlights the importance of outcome selection in balance research. While VR appears to enhance functionally relevant tasks requiring movement coordination and anticipatory control, its benefits in static postural control remain uncertain. The findings support the clinical use of VR as an adjunct to traditional balance training, particularly in programs aiming to improve dynamic and functional balance.

## 8. Conclusions

VR-based training appears to be more effective than control in improving dynamic and functional—but not static—balance outcomes in individuals with CLBP, particularly in measures such as the TUG, SEBT, and the functional reaching test. This review provides the first systematic synthesis and meta-analysis of RCT evidence highlighting the role of VR-based training in balance rehabilitation for individuals with CLBP. Further rigorous research with longer interventions, larger study samples, and extended follow-up is needed to confirm and strengthen these findings.

## Figures and Tables

**Table 3:** GRADE Summary of Findings: VR-based training vs. Control for Balance Outcomes.

Outcome	Studies (*n*)	Effect (MD, 95% CI)	I^2^	Certainty (GRADE)	Reasons for Downgrading
Single-Leg Stance (s)	3 RCTs (71)	+0.67 (−2.78 to 4.11)	21%	Low	Imprecision (−1); Risk of Bias (−1)
CoP Medio-Lateral Displacement (mm)	2 RCTs (51)	+1.42 (−1.43 to 4.27)	96%	Very low	Inconsistency (−1/−2); Imprecision (−1); Risk of Bias (−1)
CoP Velocity (mm/s)	2 RCTs (51)	+1.25 (−1.60 to 4.10)	95%	Very low	Inconsistency (−1/−2); Imprecision (−1); Risk of Bias (−1)
Timed Up & Go (s)	2 RCTs (68)	−2.29 (−2.91 to −1.66)	0%	Moderate	Risk of Bias (−1)
Dynamic Balance in Reaching (cm)	2 RCTs (61)	+7.80 (2.08 to 13.52)	0%	Moderate	Risk of Bias (−1)

MD; mean difference, CI; confidence interval, RCT; randomized controlled trial.

## Data Availability

All data analyzed in this study are included in the published articles cited in the systematic review. No new primary data were generated.

## Supplementary Materials

The following supporting information can be downloaded at: https://www.mdpi.com/article/10.3390/jcm14207247/s1, Supplementary File S1. Search strategy. Supplementary File S2. Excluded studies. Supplementary File S3. Clinicaltrials.gov excluded studies.

## Abbreviations

The following abbreviations are used in this manuscript:

CLBP	Chronic Low Back Pain
VR	Virtual Reality
RCT	Randomized Controlled Trial
TUG	Timed Up and Go (test)
SLS	Single Leg Stance (test)
CoP	Center of Pressure
LoS	Limits of Stability
SEBT	Star Excursion Balance Test
PEDro	Physiotherapy Evidence Database
PRISMA	Preferred Reporting Items for Systematic Reviews and Meta-Analyses
PROSPERO	International Prospective Register of Systematic Reviews
EG	Experimental Group
CG	Control Group
NWE	Nintendo Wii Exercise
LSE	Lumbar Stabilization Exercise
MCE	Motor Control Exercise
ADIM	Abdominal Drawing-In Maneuver
AT	Activation Time
APA	Anticipatory Postural Adjustment
CPA	Compensatory Postural Adjustment
TrA	Transversus Abdominis
TA	Tibialis Anterior
MF	Multifidus
IEMG	Integral of Electromyography
SD	Standard Deviation
MD	Mean Difference
CI	Confidence Interval
I^2^	I-squared statistic (for heterogeneity)
NIA	No Information Available
EA	Elliptical Area
IQR	Interquartile Range
MT	Magnetic Therapy
COG	Center of Gravity
IV	Inverse Variance
