# Supplementary material for: Virtual Reality Training for Balance in Patients with Chronic Low Back Pain: A Systematic Review and Meta-Analysis
Source: J Clin Med. 2025 Oct 14;14(20):7247. doi: 10.3390/jcm14207247 (PMC12564781; doi:10.3390/jcm14207247)
Supplement: Supplementary file 1 [file jcm-14-07247-s001.zip › jcm-3821210-supplementary.pdf]

Supplementary Files

Supplementary file # 1

Search Strategy

1. Database: PubMed

Date	01/07/2024
Filters	None
Keywords	("Virtual Reality" [Mesh] OR "Virtual reality training" OR VR* OR “Wii” OR “virtual walking” OR “skateboarding*” OR “GAM*” OR “Electronic Game“ OR "VR training" OR "VR learning" OR "Artificial intelligence training" OR "Immersive training" OR "Virtual simulation training" OR "3D training" OR "Digital training" OR "Augmented Reality" [Mesh] OR "Augmented reality training" OR "Mixed reality training" OR "Interactive training") AND ("Low Back Pain" [Mesh] OR "Chronic low back pain" OR "CLBP" OR "Persistent low back pain" OR "Recurrent low back pain" OR “lumbar” OR "Persistent backache" OR "Intractable back pain" OR "Chronic lumbago") AND ("Postural Balance" [Mesh] OR Balance OR Stability OR Equilibrium OR Coordination OR "Postural control" OR Steadiness OR Poise OR Symmetry OR "Center of gravity*" OR "Body control*" OR Proprioception OR "Center of Pressure*" OR "Centre of Pressure*")

2. Database: Web of Science

Date	01/07/2024
Filters	- Document Type: Article<br>- Languages: English<br>- Research Areas: Rehabilitation<br>- Research within all fields: Randomized Controlled Trial
Keywords	(TS=("Virtual Realit*" OR VR* OR "Virtual reality training" OR "VR training" OR "VR learning" OR “Artificial intelligence training" OR "Immersive training" OR "Virtual simulation training" OR "3D training" OR "Digital training" OR "Augmented reality training" OR "Mixed reality training" OR "Interactive training") AND TS=("Chronic low back pain" OR "CLBP" OR "Persistent low back pain" OR "Recurrent low back pain" OR "Persistent backache" OR "Intractable back pain" OR "Chronic lumbago") AND TS=(“Balance” OR “Stabilit”* OR “Equilibrium” OR “Coordination” OR “Postural control” OR “Steadiness” OR “Poise” OR “Symmetr*” OR “Center of gravit*” OR “Body control*” OR “Proprioception” OR “Center of Pressure*” OR “Centre of Pressure*”))

3. Database: Medline

Date	01/07/2024
Filters	- Journal Article<br>- Randomized Controlled Trial<br>- Humans<br>- English
Keywords	(TS("Virtual Realit*" OR VR* OR "Virtual reality training" OR "VR training" OR "VR learning" OR “Artificial intelligence training" OR "Immersive training" OR "Virtual simulation training" OR "3D training" OR "Digital training" OR "Augmented reality training" OR "Mixed reality training" OR "Interactive training") AND TS=("Chronic low back pain" OR "CLBP" OR "Persistent low back pain" OR "Recurrent low back pain" OR "Persistent backache" OR "Intractable back pain" OR "Chronic lumbago") AND TS=(Balance OR Stabilit* OR Equilibrium OR Coordination OR “Postural control” OR Steadiness OR Poise OR Symmetr* OR “Center of gravit*” OR “Body control*” OR Proprioception OR “Center of Pressure*” OR “Centre of Pressure*”))

4. Database: Scopus

Date	01/07/2024
Filters	- Journal Article<br>- Randomized Controlled Trial
Keywords	(TITLE-ABS-KEY ("Virtual Realit*" OR VR* OR "Virtual reality training" OR "VR training" OR "VR learning" OR “Artificial intelligence training" OR "Immersive training" OR "Virtual simulation training" OR "3D training" OR "Digital training" OR "Augmented reality training" OR "Mixed reality training" OR "Interactive training") AND TITLE-ABS-KEY ("Chronic low back pain" OR "CLBP" OR "Persistent low back pain" OR "Recurrent low back pain" OR "Persistent backache" OR "Intractable back pain" OR "Chronic lumbago") AND TITLE-ABS-KEY (Balance OR Stability OR Equilibrium OR Coordination OR “Postural control” OR Steadiness OR Poise OR Symmetry OR “Center of gravit*” OR “Body control*” OR Proprioception OR “Center of Pressure*” OR “Centre of Pressure*”))

5. Database: Dimensions

Date	01/07/2024
Filters	- Publication Type: Article<br>- Fields of Research: Allied Health and Rehabilitation<br>- Free text in full data

Keywords	("Virtual Reality" OR VR OR "Virtual reality training" OR "VR training" OR “Wii” OR "VR learning" OR “Artificial intelligence training" OR "Immersive training" OR "Virtual simulation training" OR "3D training" OR "Digital training" OR "Augmented reality training" OR "Mixed reality training" OR "Interactive training") AND ("Chronic low back pain" OR "CLBP" OR "Persistent low back pain" OR "Recurrent low back pain" OR "Persistent backache" OR "Intractable back pain" OR "Chronic lumbago") AND (Balance OR Stability OR Equilibrium OR Coordination OR “Postural control” OR Steadiness OR Poise OR Symmetry OR “Center of gravity” OR “Body control” OR Proprioception OR “Center of Pressure” OR “Centre of Pressure”)

6. Database: Semantic Scholar

Date	01/07/2024
Filters	None
Keywords	("Virtual Reality" OR VR OR "Virtual reality training" OR "VR training" OR “Wii” OR “Artificial intelligence training" OR "Immersive training" OR "Virtual simulation training" OR "3D training" OR "Augmented reality training") AND ("Chronic low back pain" OR "CLBP" OR "Persistent low back pain" OR "Recurrent low back pain" OR "Persistent backache" OR "Chronic lumbago") AND (Balance OR Stability OR Equilibrium OR Coordination OR “Postural control” OR “Center of gravity” OR “Body control” OR Proprioception OR “Center of Pressure” OR “Centre of Pressure”)

7. Database: ProQuest

Date	01/07/2024
Filters	- Scholarly Journals<br>- Article<br>- English<br>- NOT Sci Tech Premium Collection
Keywords	("Virtual Reality" OR VR OR "Virtual reality training" OR "VR training" OR “Wii” OR “Artificial intelligence training" OR "Immersive training" OR "Virtual simulation training" OR "3D training" OR "Augmented reality training") AND ("Chronic low back pain" OR "CLBP" OR "Persistent low back pain" OR "Recurrent low back pain" OR "Persistent backache" OR "Chronic lumbago") AND (Balance OR Stability OR Equilibrium OR Coordination OR “Postural control” OR “Center of gravity” OR “Body control” OR Proprioception OR “Center of Pressure” OR “Centre of Pressure”)

8. Database: Clinicaltrial.gov

Date	17/09/2025
Filters	None
Keywords	("Virtual Reality" OR VR OR "Virtual reality training" OR "VR training" OR "Wii" OR "Artificial intelligence training" OR "Immersive training" OR "Virtual simulation training" OR "3D training" OR "Augmented reality training") AND ("Chronic low back pain" OR CLBP OR "Persistent low back pain" OR "Recurrent low back pain" OR "Persistent backache" OR "Chronic lumbago") AND (Balance OR Stability OR Equilibrium OR Coordination OR "Postural control" OR "Center of gravity" OR "Body control" OR Proprioception OR "Center of Pressure" OR "Centre of Pressure")

Supplementary file # 2

Excluded Studies

No.	Study Title	Reason for Exclusion	Author/ Year
1.	The effects of exergaming on pain, postural control, technology acceptance and flow experience in older people with chronic musculoskeletal pain: a randomized controlled trial	Not relevant (population is not limited to LBP)	Ditchburn 2020
2.	Efficacy of Nintendo Wii training on mechanical leg muscle function and postural balance in community-dwelling older adults: a randomized controlled trial	Not relevant (population is not limited to LBP)	Jorgensen 2013
3.	Radiological (Magnetic Resonance Image and Ultrasound) and biochemical effects of virtual reality training on balance training in football players with chronic low back pain: A randomized controlled study.	No balance outcome measure	Nambi 2021
4.	Effects of Nintendo Ring Fit Adventure Exergame on Pain and Psychological Factors in Patients with Chronic Low Back Pain	No balance outcome measure	Sato 2021
5.	Improvements in Dynamic Balance Using an Adaptive Snowboard with the Nintendo Wii	Pedro score < 6	Sullivan 2012
6.	Video-Game–Based Exercises for Older People with Chronic Low Back Pain: A Randomized Controlled table Trial (GAMEBACK(	No balance outcome measure	Zadro 2018
7.	Video-Game-Based Exercises for Older People with Chronic Low Back Pain: A Randomized Controlled table Trial (GAMEBACK(	Duplicate	Zadro 2019
8.	Use of the CAREN system as a treatment adjunct for Canadian Armed Forces members with chronic non-specific low back pain: a pilot study	Pedro score < 6	Hebert, -2015
9.	The efficacy of a HUBER exercise system mediated sensorimotor training protocol on proprioceptive system, lumbar movement control and quality of life in patients with chronic non-specific low back pain	Pedro score < 6	Letafatkar, 2017
10.	Virtual reality skateboarding training for balance and functional performance in degenerative lumbar spine disease	Pedro score < 6	Tsai, 2024

Supplementary file # 3

Clinicaltrials.gov Excluded Studies

No.	NCT Number	Study Title	Study Status	Exclusion Reason
1.	NCT06030960	Effects of Virtual Reality Stabilization Training in Patients with Low Back Pain	COMPLETED	No published results
2.	NCT07172828	Virtual Reality-Based Rehabilitation in Chronic Low Back Pain: Effects on Muscle Architecture, Balance, and Satisfaction	NOT_YET_RECRUITING	No published results
3.	NCT06757413	Virtual Reality Programme Incorporating Psychology and Physiotherapy for Chronic Low Back Pain	COMPLETED	No balance outcome measure
4.	NCT04236804	VANISH for Chronic Low Back Pain	COMPLETED	No balance outcome measure
5.	NCT05253599	Radiographic and Inflammatory Biomarker Changes in Chronic Low Back Pain	COMPLETED	No balance outcome measure
6.	NCT03987334	Virtual Reality Rehabilitation in Neck Pain Subjects	UNKNOWN	Wrong population
7.	NCT05423626	Innovative Technologies in Restoring Gait and Balance Functions in Ischemic Stroke Patients at the Inpatient Stage	UNKNOWN	Wrong population
8.	NCT01503203	Physical Virtual Training for Older Women	UNKNOWN	No published results reported on clinicaltrials.gov but the study was already included as Monteiro-Junior et al. 2015 from other included databases.
9.	NCT06159413	LUMBAR MULTIFIDUS MUSCLES FUNCTION AMONG PATIENTS WITH LOW BACK PAIN: VIRTUAL REALITY VERSU VERSUS STABILIZATION EXERCISES.	COMPLETED	PEDro score < 6
10.	NCT04576611	LOw Level of Activity (LOLA): Education and Exercise-based Intervention for Low Back Pain	COMPLETED	No published results reported on clinicaltrials.gov but was published as: Sitges et al. 2022, without a balance outcome measure.
